# Retreading of Tyres

**Published:** 1911-03-25

**Authors:** 


### Motoring Notes.

### RETREADING OF TYRES.

The question is often asked, "Does it pay to retread motor tyres?" The answer to this must be "Yes, unquestionably," if the retreading is done in time. Spring is now approaching, and is a suitable time to have this desirable repair effected. Most medical men have run their cars continuously through the winter, with the result that not only have the treads of the tyres become thinned, but, owing to wet having found its way past the rims, the canvas has probably suffered to some slight extent.

The cost of retreading is approximately half the cost of a new cover (depending of course upon the amount of deterioration of the cover), and the latter ought to be good for another 3,000 to 4,000 miles, whereas if the cover is run until the canvas is exposed, not only is it worthless for retreading, but a burst is very liable to occur, with consequent destruction of the inner tube. By dismounting the cover, and feeling with two fingers, one placed inside the cover and the other outside, the thickness can be approximately gauged.

### Advantages.

In the case of my own car, I had fitted originally four grooved Dunlop tyres, 760 mm. × 90 mm. The car has now run 4,100 miles over all kinds of roads, and throughout the winter. By running the tyres to destruction I can get about another 1,500 miles out of them, and then would have to renew the four at a cost of about £23, with the addition probably of a new tube or two. Instead of waiting for this, I have purchased a new cover at a cost of £5 12s., which I have had fitted to the back wheel. The removed cover is being repaired and retreaded at a cost of £2 6s., and will then replace one of the front tyres, which will go for similar repairs. The cover which has been on my Stepney wheel and never used replaces the other cover on my back wheel.

Thus I have two new covers on the back, and two retreaded covers on the front wheels, which ought to carry me through the coming summer and the following winter.

To spread the cost over a longer period, I do not intend to have the two old covers which I now have retreaded. One will go on the Stepney wheel, and the other kept as a spare, both to be retreaded next year.

### Cost.

The cost works out as follows: One new cover, £5 12s., and two retreaded tyres, £4 12s. = £10 4s., as against four new covers in a short time at a cost of £23 to £25. This is a saving of £13 to £14 on one's annual tyre bill—an item worth considering—and, in addition, I have now a spare cover which I did not possess before.

In connection with the care of tyres, I attribute my freedom from punctures (one in 4,100 miles) to frequent examination of the tyres on long journeys at every stoppage on the road, and removal of nails, flints, etc.

### Tyre Saving.

Tyres can be saved considerably by careful driving. In turning corners the clutch should be thrown out and the car allowed to go round by its own momentum. Never change speed from high to low gear until the car is travelling at the normal speed for which that gear is suited, otherwise a braking effect is produced and the tyre scraped on the ground. Do not pull the car up too close to the curb, because the tyre can be seriously damaged at its thinnest part by scraping against the kerbstone. Each day have mud and grease (especially grease) removed from the tyres, and the tyres then dried.

